# Umbilical Myiasis by *Cochliomyia hominivorax* in an Infant in Colombia

**DOI:** 10.3389/fmed.2019.00292

**Published:** 2020-01-22

**Authors:** Juan David Ruiz-Zapata, Luis Mauricio Figueroa-Gutiérrez, Jaime Alberto Mesa-Franco, Paula Andrea Moreno-Gutierrez

**Affiliations:** ^1^Faculty of Health Sciences, Universidad Tecnológica de Pereira, Pereira, Colombia; ^2^Grupo de Investigación en Biomedicina, Fundación Universitaria Autónoma de las Américas, Pereira, Colombia

**Keywords:** screwworm infection, newborn, umbilicus, myiasis, ivermectin, Colombia

## Abstract

Myasis is the infestation by fly larvae (Diptera) in live vertebrates including humans. Myasis has been reported most commonly in tropical and subtropical areas around the world with poor sanitation and presence of cattle. Neonatal umbilical myiasis is an important cause of death in bovines and produces major economic losses in the livestock industry. However, its presentation in humans is rare, with a few cases reported worldwide. Moreover, umbilical myasis can be life-treating due to the risk of larvae migration to deeper tissues of the abdomen, omphalitis, and sepsis. We describe the case of a 7-day-old infant admitted to the hospital due to umbilical cord myiasis. In total, 55 larvae were removed from the wound and identified as *Cochliomyia hominivorax*. The patient recovered satisfactorily after treatment with ivermectin and amoxicillin. A literature search was performed in Pubmed, Medline, Lilacs and Google Scholar, with 64 cases of myasis by *C. hominivorax* being reviewed. Oral cavity, wounds, scalp and natural orifices are the main affected anatomical areas. Risk factors include the extremes of age, male sex, poor hygiene, alcohol and drug use, cancer, and mental disability. Programs for human myiasis prevention and surveillance are needed in neotropical areas where living conditions make it difficult to implement control strategies.

## Introduction

Myiasis is infestation by fly larvae (Diptera) in live vertebrates, including humans. Fly larvae feed on wound tissue of their host, causing a disease whose severity may depend on the larva species and anatomical sites affected (1, 2). It is widespread in neotropical areas around the world, causing economic and public health problems in low-income populations. Human infection is facilitated by poor hygienic conditions and close contact with wild or domestic animals (2, 3). Umbilical cord myiasis is a common type of wound myiasis in animals but it has been described only rarely in humans (2). We present the first report of neonatal umbilical myiasis in Colombia and review the most relevant aspects of this disease. Recent case reports of myiasis by *C. hominivorax* are reviewed in the discussion.

## Case Report

The research procedures for this case were carried out in accordance with the recommendations of the guidelines of the Helsinki Committee. Written informed consent was obtained from the mother of the newborn for pictures and publication of this case.

A 7-day-old female neonate was taken to a primary health facility in June 2017 because something was coming out of her umbilicus. The patient was born full-term at the local hospital by vaginal delivery from a 17-year-old mother. At birth, the newborn had respiratory depression and mild perinatal asphyxia but without further complications. The umbilical cord was cut following standard care measures for in-hospital delivery. The patient lived on a farm with a cowshed next to the house in the rural area of the municipality of La Virginia (04°54′1.617″ N, 75°52′47.445″ W), in the state of Risaralda, located in the coffee region of Colombia. The mother practiced exclusive breastfeeding and used a fabric girdle, which is traditionally used in Colombia for protection of the umbilical stump during the first days of life.

The neonate was transferred to a tertiary care hospital. On admission, she was visibly irritated and jaundiced. On physical exam, weight was 3,300 g and vital signs (temperature, heart rate, respiratory rate, blood pressure, and blood oxygen levels) were normal. Umbilical stump inspection revealed numerous live larvae (Supplementary Figure 1) and foul-smelling serohaematic secretion. The rest of the examination was normal. Initial blood count showed 20,140 leukocytes/μl (52% neutrophils, 3% eosinophils, 4% lymphocytes, and 5% monocytes). Total serum bilirubin was 18.0 mg/dl (cutoff point to consider phototherapy: 20.5 mg/dl) (4). Wound and blood cultures on admission and 48 h later were negative.

Initial treatment included covering the umbilical stump with gauze soaked in ivermectin and nitrofurazone, followed by a single oral dose of ivermectin (0.15 mg/kg). To prevent late-onset sepsis, intravenous ampicillin (200 mg/kg/day) and gentamicin (4 mg/kg/day) were administered. On the second day, 39 live larvae were removed from the umbilical stump under aseptic conditions using a surgical clamp. One live and 15 dead larvae were extracted on the third day. A follow-up abdominal ultrasonography was normal and the patient was discharged 7 days after admission.

After extraction, seven larvae were preserved in a solution containing 80% alcohol. The specimens were sent to an entomologist and examined using a microscope with 10× magnification. Third instar larvae of *C. hominivorax* were identified by their smooth appearance with prominent spine bands and one body process in the last segments (Supplementary Figure 2). Pigmented dorsal tracheal trunks were present in two to three of the last segments. The posterior spiracular plates contained three oval-shaped slips pointing to the peritreme (5).

## Discussion

Umbilical myiasis is a rare type of wound myiasis in humans, but the occurrence of cases in widely distributed areas shows that this may be a latent risk in all neotropic zones were myiasis has been reported (2). A handful of case reports of umbilical myiasis have been made, mainly in India (3, 6–13). One case was reported in the United States (14) and another in Argentina (15), the latter associated with *C. hominivorax*. The largest case collection of umbilical myiasis was carried out in Nigeria, where active detection in a region of the Niger Delta resulted in 55 cases of omphalitis (16). Other anatomical sites of myiasis in human neonates include the nostrils (17), ear (18), skin (19), and genitals (20).

The warm and moist environment of the umbilical stump attracts the female flies to lay their eggs on it (11). In our case, the use of an umbilical girdle could have retained moisture around the stump and delayed the separation, creating ideal conditions for larvae growth and also hiding the disease. Umbilical girdles were used traditionally to secure the navel of newborns (21) and remain a common practice in Colombia that goes against current recommendations to keep the stump uncovered to help dry out the base. The girdle also facilitates omphalitis, which in turn increases the size of the wound and creates a proper environment for egg hatching (11). Traditional methods for stump care, such as application of cow dung or herb leaves on the umbilicus of neonates, have been described in previous reports as sources of cross-contamination (16, 22).

Clinical signs of umbilical myiasis are hardly recognized by the caregiver. The disease is usually detected once the larvae are visible or clinical signs of omphalitis appear (11). Imaging and biopsy are rarely necessary for diagnosis but may be useful in umbilical myiasis to determine the extent of the infestation and any organ involvement. Leukocytosis along with neutrophilia and eosinophilia are common clinical findings (2). Hyperbilirubinaemia that resolved after larvae extraction was reported in one case of cutaneous myiasis by Drosophila in a newborn (23), but not in prior cases of neonatal umbilical myiasis.

Neonatal myiasis has been consistently attributed to conditions related to low socioeconomic status, such as poor hygiene, contact with farm animals, home delivery using unsterilized instruments and the use of traditional methods to take care of the stump (8, 9, 11, 24). Nonetheless, wound myiasis can also be an indicator of neglect or self-neglect (24). Thus, social counseling should be considered in these cases and newborn care must be reinforced. Adequate wound care, keeping the umbilicus covered with clean dressings and adequate hygienic habits in general should all be included in the recommendations given to the mother or caretaker before discharge (2, 14, 15).

The New World screwworm (*C. hominivorax*), is the most common species causing myiasis in Central and South America. The incidence of human myiasis by this species has been declining progressively since 1958 due to the implementation of programmes using the sterile insect technique (SIT) that have led to the eradication of *C. hominivorax* in Curacao, North and Central America and North Africa (25). Sixty-five case reports of human disease have been published from 2000 up to 30 September 2019 according to a literature search performed in Pubmed, Medline, Google Scholar and Lilacs (Table 1). Sixty of the cases (92%) occurred in South America, mainly in Brazil (*n* = 31, 48%) and Argentina (*n* = 7, 11%). There was one case report in India, but the species could have been mistakenly identified. Common anatomical sites of infection were the oral cavity, chronic or traumatic wounds, scalp and natural orifices (ear, nose, vagina). Risk factors for infection include the extreme ages, male gender, rural residency, poor hygienic conditions, cancer, alcohol and drug use, malnutrition, mental impairment, prolonged mouth opening, and prosthetic material. Myiasis in the scalp was facilitated by pediculosis or seborrheic dermatitis.

**Table 1:** Cases of myiasis by *Cochliomyia hominivorax* published since 2000 in Pubmed, Medline, Google Scholar, and Lilacs.

**Country**	**Age and sex**	**Location**	**Risk factors**	***n* larvae**	**References**
Chile	37 M	Ear	Travel	22	(26)
Brazil	17 F	Vulva	Pregnancy, condilomatosis	67	(27)
Brazil	8 M	Oral cavity	Leukoderma, oral breathing	19	(28)
Brazil	66 F	Oral cavity	Alcohol abuse	40	(29)
Argentina	36 M	Scalp	Poor higiene conditions, pediculosis	>40	(30)
French Guiana	70 M; NA; 40 M; 72 M; NA	Oral cavity; wound in toe; thigh ulcer; low limb ulcer; scalp	NA; Alcoholism; Ulceration; Ulceration; Pediculosis	NA	(31)
Brazil	77 F	Vulva	Mental disability, lack of social support	50	(32)
Brazil	41 M	Wound in dorsal antebrachium	Wound, adventure sports	1	(33)
Argentina	10 M	Eye protesis	Hydroxyapatite implant	20	(34)
Surinam	51 M	Ankles	Ulcer	>100	(35)
Brazil	80 M	Eye	Alcohol and tabaco abuse, lack of social support	NA	(36)
Venezuela	40 F	Thigh ulcer	Bedridden, epilepsy	20	(37)
French Guiana	84 M	Nose wound	Hospitalized	9	(38)
Brazil	87 F	Vagina	Obese, diabetic, hypertensive, low socio-economic status	NA	(39)
Brazil	55 M	Rhino-orbital area	Ethmoidal sinus carcinoma	NA	(40)
Colombia	79 M	Skin carcinoma in the eye orbit	Skin carcinoma in the eye orbit	NA	(41)
Brazil	27 F	Eye	NA	1	(42)
Cuba	60 M	Nasal tumor	Nasal tumor	>200	(43)
Brazil	63 M	Pharynx and esophagus	Mouth-breather	100	(44)
Argentina	58 M	Scalp and brain cavity	Tuberculosis	NA	(45)
Brazil	7 F	Periorbital	Cerebral palsy	NA	(46)
Argentina	11-day old	Umbilical stump	Newborn	23	(15)
India	46 M	Facial wound	Poor higiene conditions, low IQ	NA	(47)
Colombia	12 F	Scalp	Psoriasis	142	(48)
Brazil	22 M; 70 M	Wound from dental extraction; Palate	Wound from dental extraction, mental disability; Senile	24; NA	(49)
Brazil	30 M	Scalp	Homeless, smoker, drug user	518	(50)
Brazil	5 F	Oral cavity	Poor oral hygiene	2	(51)
Brazil	89 F	Uterine prolapse	Dementia, poverty	NA	(52)
Venezuela	32 M	Pin-site	Alcohol and drug abuse, external metalic bone fixator	105	(53)
Brazil	9 NA	Oral cavity	Poor oral hygiene, malnutrition	NA	(54)
Colombia	80 F	Nose	Malnutrition, nasal septum perforation	NA	(55)
Brazil	80 M	Orbital region	Rural area, living alone	NA	(36)
Brazil	72 M; 35 F	Oral cavity; periodontal area	Hospitalized; Alcohol consumption	NA	(56)
Colombia	7 F	Scalp	Poor higiene conditions, pediculosis	NA	(57)
Argentina	32 M	Wound in scalp	Drug user	71	(58)
Peru	62 M	Oral cavity	Parkinson	75	(59)
Brazil	49 M	Tracheostomy site	Alcohol and tabaco abuse, larynx cancer, poor hygiene conditions	20	(60)
Cuba	83 F; 87 M	Facial skin carcinoma; facial skin carcinoma	Alzheimer's, rural residency, skin carcinoma; Skin carcinoma	NA; NA	(61)
Argentina	11 M; 9 F	Ear	NA; Malnutrition, intestinal parasitosis	NA	(62)
North India	80 M	Wound in eyelid skin	Squamous cell carcinoma	NA	(63)
Haiti	16 F; 10 M	Wound in eye; facial wound	Earthquake victims	3 7	(64)
Brazil	97 M	Oral cavity	Multiple diseases, Bedridden	110	(65)
Brazil	22 cases between 2007 and 2008	Mostly open wounds	Age group 41–50 years old, black race, low level of education, low hygiene conditions and poor urban infrastructure	NA	(1)
Brazil	49 M	Thoracic cavity	Hospitalized, tracheostomy	32	(66)
Brazil	54 M	Oral cavity	Aphasia	NA	(67)
Colombia	50 M; 29 M; 20 M; 35 M; 6 M; 58 M	Oral cavity	Craniofascial trauma, altered conciousness	30; 60; 39; 126; 105;81	(68)
Brazil	10 cases between 2005 and 2011	Oral or maxillofacial	Diabetes, mental disease, AIDS, mental impairment, depression	NA	(69)
Brazil	95 M	Oral cavity	Hospitalized	103	(70)
Brazil	59 M	Wound in shoulder	Wound	287	(71)
Brazil	38 M	Mouth	Trauma	55	(72)
Argentina	54 M	Diabetic food ulcer	Diabetic food ulcer	NA	(73)
Brazil	36 M	Oral cavity	Leukoderma, rural residency	75	(74)
Colombia	26 M	Pin-site	External metalic bone fixator	80	(75)
Dominican Republic	26 F	Ear	Alcohol consumption, travel	NA	(76)
Ecuador	24 F	Oral cavity	Brain damage, prolonged mouth opening	NA	(77)
Peru	67 M	Tracheostomy site	Tracheostomy, gastrostomy, esophageal cancer	NA	(78)
Peru	9-F	Scalp	Pediculosis	42	(79)
Brazil	22 M; 50 F; 45 F; 33 M; 26 M; 57 M; 21 F; 24 M; 65 M	Head and neck	Poor oral hygiene, trauma	NA	(80)
Brazil	41 F	Breast	Breast cancer	NA	(81)
Chile	26-F	Scalp	Seborrheic dermatitis	29	(82)
Brazil	41 F	Finger	Necrosis and amputation	132	(83)
Brazil	27 M	Scalp	Mental disability	27	(84)
Peru	71 M; 71 F; 67 F; 85 M; 73 F	Foot; nose; nose; breast	Skin eruption; Cellulite; Necrosis; Ulcera	NA	(85)
Colombia	77 M	Pin-site	Prosthetic material, chronic wound	100	(86)

*NA, not available; F, female; M, male*.

In Colombia, the geographic distribution and economic burden of *C. hominivorax*, as well as the epidemiology of myiasis in both animals and humans, is unknown but this species is recognized as in important cause of livestock loss (87). Human myiasis by this species has been reported in the states of Antioquia (88, 89), Atlantico (57), Cundinamarca (41), and Boyaca (90), however notification of cases is not mandatory. Research is needed on the biology, epidemiology and population dynamics of this species in order to assess the political, geographic and economic viability of the implementation of programs for insect control in the country (87). Thus, nationwide protocols and surveillance systems are urgently needed to control this ongoing threat to animal and human health.

During its larvae stage, *C. hominivorax* is an obligate parasite of warm-blooded animals, including humans. Once the female is gravid, it deposits an average of 200 eggs in open wounds or natural orifices (1). Egg hatching occurs in approximately 12 h and then it takes 5–7 days for larvae to reach the third instar of maturity inside bovine wounds. This means that the patient possibly was infected in the first 2 days of life. Larvae penetrate deeply into wounds, tearing tissue and making tunnels with their mouths to find a warm and moist place. Then, they hook and cause an extensive destruction of tissue known as traumatic myiasis, which provokes wound swelling that may facilitate bacterial infection (91). Umbilical myiasis is particularly dangerous because it might induce fistulation, penetration of deep layers of the abdomen wall and secondary sepsis associated with omphalitis (2, 92), although none of these were found in our patient.

As in our case, treatment of myiasis is based on the removal of all visible larvae, cleaning of the wound and debridement of remaining necrotic tissue. Irrigation is helpful if the lesions have holes and/or cavities. Local application of ivermectin paralyzes the parasite and kills the larvae, facilitating the extraction and relieving pain (31). Turpentine or ether is used to suffocate the larvae, but this practice is not recommended as it could lead to complications such as anaphylaxis and sepsis (22). Surgical treatment is required when larvae are dead, decomposing or laying in deep tissues (8). Topical anthelmintic medication, bactericides, tetanus toxoid vaccine and systemic antibiotics should also be considered to prevent secondary sepsis. In many reports, the use of systemic ivermectin showed positive results, but further studies are needed to consider this a standard therapy (2, 7, 8).

Correct identification by a trained entomologist is helpful to understand the infestation mechanism, to plan treatment and to consider preventive actions. For etiological diagnosis, the larvae should be immersed in hot water for 30 s to retain length and morphology and then preserved in a 70–90% ethanol solution or isopropyl alcohol. The regions where the patient has been, the climatic conditions and the endemic species are also important for accurate identification (2). The peak period of infestation by *C. hominivorax* has been reported to be between June and August, in humid and warm locations (5), such as the city where the patient lived.

Livestock is an important economic source in neotropical regions where poverty and inadequate health conditions make it difficult to implement control and eradication programs. Therefore, myiasis will continue to be a sanitary problem in many countries of America, Africa and Asia. Furthermore, global warming and internationalization are likely to influence the migration of screwworm and other myiasis-causing species into new geographic areas that were previously unaffected by this problem. Naïve livestock host are more susceptible to insect replication, increasing the likelihood of outbreaks (93). Groups of individuals at high risk of myiasis should be targeted in prevention programs for *C. hominivorax* infection in areas were insect eradication programs are not available.

## Ethics Statement

Ethical review and approval was not required for the study on human participants in accordance with the local legislation and institutional requirements. Written informed consent to participate in this study was provided by the participants' legal guardian/next of kin. A written informed consent was obtained from the mother of the newborn for pictures and publication of this case.

## Author Contributions

LF-G and JM-F contributed to the diagnosis and treatment of the patient. They also obtained informed consent and gathered clinical data. JR-Z and PM-G reviewed the literature and wrote the manuscript. All the authors discussed and analyzed the case.

### Conflict of Interest

The authors declare that the research was conducted in the absence of any commercial or financial relationships that could be construed as a potential conflict of interest.
