# Supplementary material for: Umbilical Myiasis by Cochliomyia hominivorax in an Infant in Colombia
Source: Front Med (Lausanne). 2020 Jan 22;6:292. doi: 10.3389/fmed.2019.00292 (PMC6987426; doi:10.3389/fmed.2019.00292)
Supplement: Supplementary file 1 [file Data_Sheet_1.docx]

Supplementary Material

# Supplementary Figures


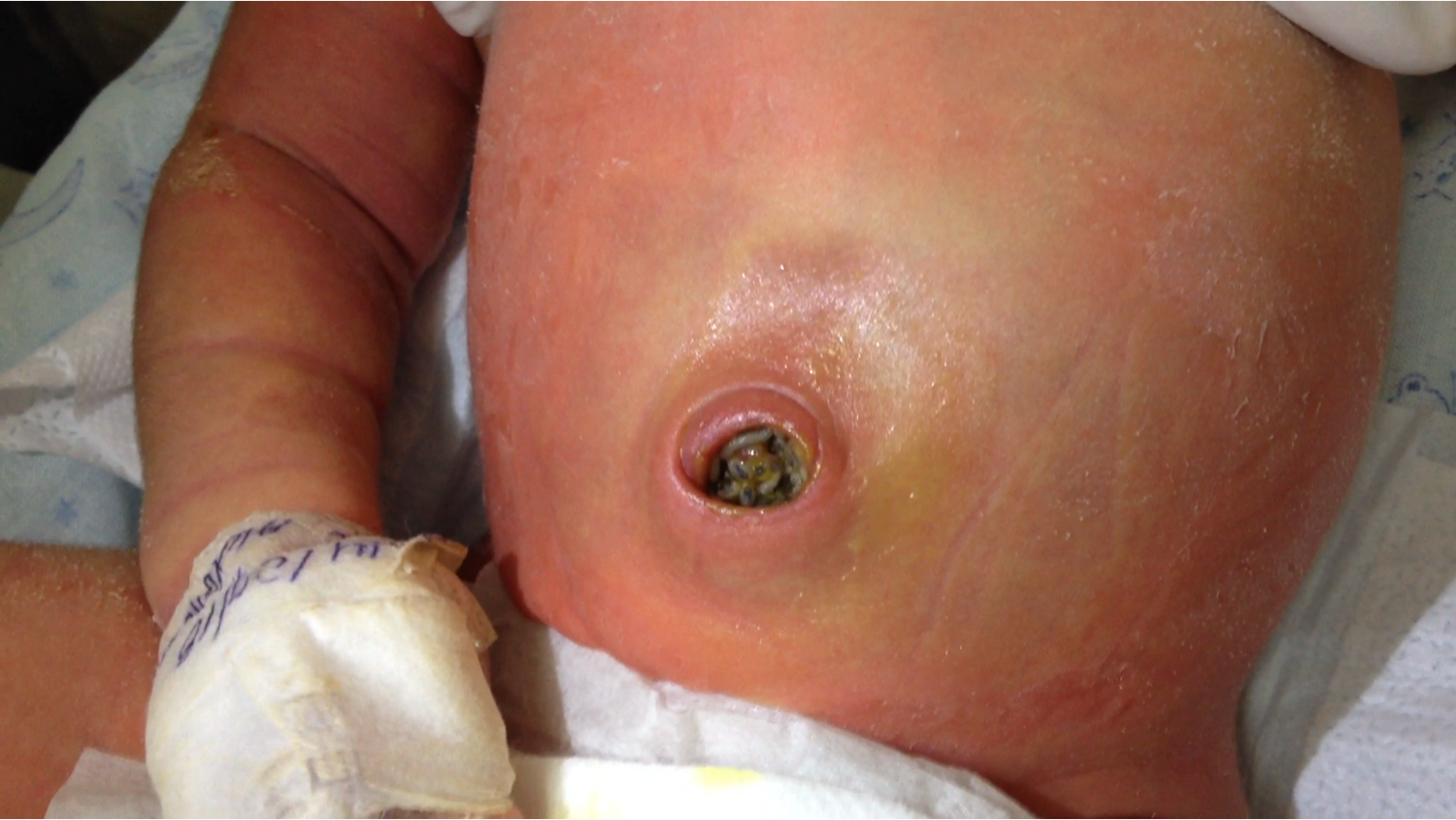


**Supplementary Figure 1.** Alive *Cochliomyia hominivorax* larvae inside the umbilical stump of a 7-day old neonate.


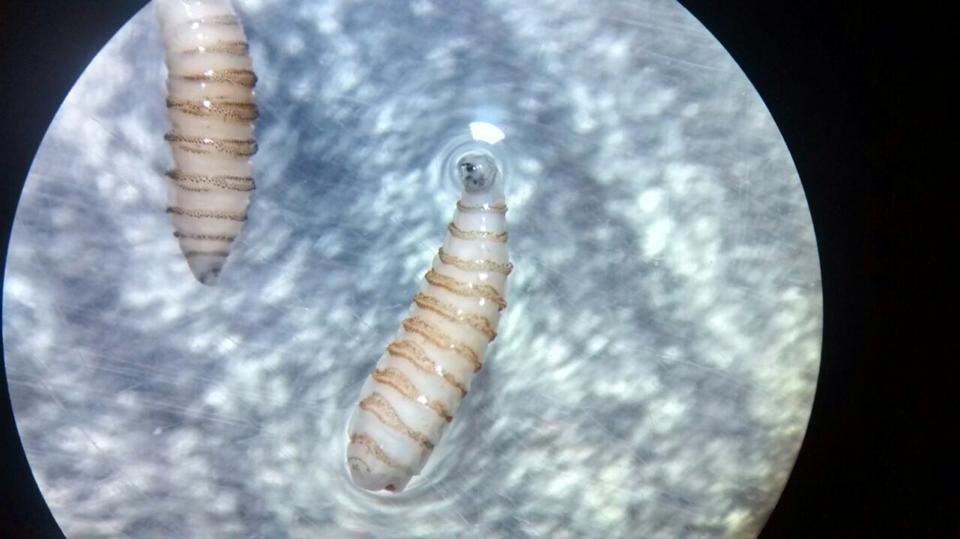


**Supplementary Figure 2.** Macroscopic view of a *Cochlyhomia hominivorax* larvae extracted to the newborn.
